# Fourth Nerve Palsy After Hair Replacement Surgery and Prolonged Vertical Position in a Patient With Mega-Cisterna-Magna: Report of a Case

**DOI:** 10.7759/cureus.95830

**Published:** 2025-10-31

**Authors:** Julio González Martín-Moro, Vicente Miralles Pechuan, Fernando Gómez Sanz, Felipe Aguilera del Hoyo, Maria Rosario Gómez de Liaño

**Affiliations:** 1 Ophthalmology, Universidad Francisco de Vitoria, Madrid, ESP; 2 Ophthalmology, Hospital Universitario del Henares, Madrid, ESP; 3 Radiodiagnosis, Hospital Universitario del Henares, Madrid, ESP; 4 Ophthalmology, Hospital Universitario Clínico San Carlos, Madrid, ESP

**Keywords:** anatomical variant, cerebrospinal fluid, fourth nerve palsy, hair transplant, intracranial pressure, isolated oculomotor nerve palsy, mega cisterna magna, oculomotor palsy, prone position, spaceflight associated neuro-ocular syndrome

## Abstract

Hair replacement surgery is a common cosmetic procedure. We present the case of a healthy 47-year-old man who suffered a right fourth nerve palsy five days after surgery. Magnetic resonance imaging revealed the presence of a mega-cisterna magna. We hypothesize that in the presence of this anatomical variant, the reduction of pressure within this space, caused by prolonged vertical posture during the postoperative period, may have led to cerebrospinal fluid shifts causing brainstem anteroposterior instability. The special configuration of the fourth cranial nerve, which curves around the brainstem, and the fact that it is very thin and long, make it particularly vulnerable to anteroposterior movements of the brainstem. Therefore, we believe that small anteroposterior displacements of the brainstem could have stretched the nerve, inducing the mild paresis we report.

## Introduction

Hair replacement surgery (HRS) is one of the most frequently performed cosmetic procedures worldwide. Local complications, such as bleeding, edema, infection, wound dehiscence, necrosis, and scarring, are relatively common. Anaphylactic shock, vaso-vagal shock, uncontrolled bleeding bronchospasm, cardiac events, angioedema, deep venous thrombosis and malignant hyperthermia are recognized as potential complications, although not usually reported in medical literature [1-6].

Transient anesthesia and dysesthesia secondary to local injury of the scalp’s sensory nerves appear to be relatively frequent minor complications. Permanent numbness however is uncommon and usually secondary to complete nerve transection of the greater occipital, lesser occipital, or auriculotemporal nerves [2,5]. In some cases, local injury may even lead to the formation of a neuroma in the affected nerve [2,5,6].In addition, an epidural abscess has been described in a patient with a history of previous craniotomy [7]. The hiccups occasionally reported by patients following this type of procedure could, to some extent, be interpreted as a neurological complication, possibly related to phrenic nerve irritation [2,3]. Nevertheless, a PubMed search using the algorithm ("hair replacement surgery" OR "hair transplant" OR "hair replacement") AND (neurological OR brain OR "cerebrovascular accident" OR ictus OR palsy) did not retrieve any case of diplopia after HRS or further reports of neurological complications. A systematic review including 2567 patients from 45 studies did not find any neurological complications beyond the local sensory changes already mentioned, that were present in only 1% of the cases [6]. However the authors of this meta-analysis suggested the presence of significant publication bias [6].

The fourth nerve is full of peculiarities. It is crossed, thin because it carries fibers for only one muscle, and long because it contours the brainstem. These features make it highly vulnerable to anteroposterior displacement of the brainstem and explain why it is frequently affected in closed head injuries [8-10].

In this article we present the case of a healthy 47-year-old man who developed a right fourth nerve palsy five days after undergoing HRS and we discuss the pathophysiological mechanism that we believe may have caused this rare complication.

## Case presentation

A 47-year-old previously healthy man underwent an uneventful HRS under local anesthesia. The procedure was performed at another center by a nurse under the supervision of a dermatologist. The patient was premedicated with 7.5 mg of midazolam. The procedure was performed under local anesthesia (in total 30 ml of 2% lidocaine, 10 ml of 0.5% bupivacaine and 2 mg of adrenaline were injected into the scalp). The patient had been on treatment with minoxidil and dutasteride for six months. A total of 4,100 follicular units were extracted from the occipital region, of which 1,500 were implanted in the crown and 2,500 in the frontal region, using a high-intensity sapphire follicular unit extraction (FUE) technique. Mesotherapy with 0.05% dutasteride was also performed intraoperatively. Antibiotic prophylaxis was carried out with 500 mg of cephalexin (for five days, starting on the day of surgery). Postoperative pain was managed with 575 mg of metamizole and 1 g of paracetamol alternately. The patient continued treatment with 0.5 mg of dutasteride every 24 hours and began taking 5 mg of minoxidil every 24 hours starting 20 days after surgery. A prostate-specific antigen (PSA) test was recommended through the patient’s general practitioner, and the result was normal.

The patient was a young, athletic male with no history of smoking, diabetes mellitus, or hypertension. His lipid profile was within reference ranges, and he was not receiving any long-term pharmacological treatment, other than minoxidil and dutasteride. He had never undergone major surgical procedures but had received dental care on several occasions without adverse reactions to local anesthetic agents.

Five days postoperatively, he reported an unusual visual disturbance that he was unable to characterize clearly. By postoperative day seven, he noted that the symptom became more pronounced when looking to the left and downward, which he described as vertical diplopia. On postoperative day 10, he sought ophthalmologic evaluation. During the initial postoperative period, the most notable alteration in his daily routine was related to posture. Because of the discomfort associated with resting the occipital region on a pillow, he spent most of his time standing or sitting and slept in an almost upright position during this period.

**Figure 1:**
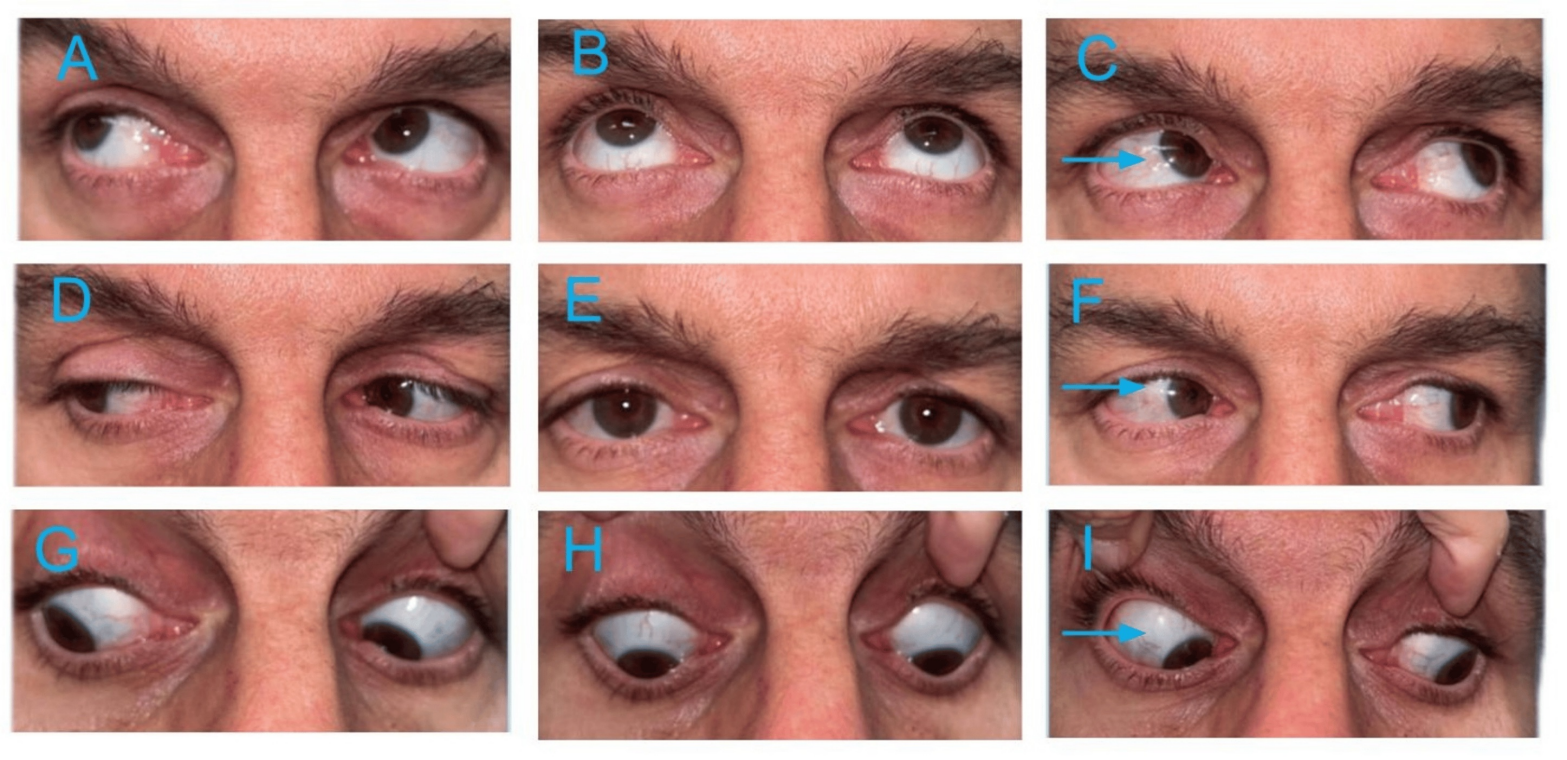
Nine-gaze positions demonstrating a small right hypertropia in left gaze. (C, F, I: blue arrow).

Visual acuity was 20/20 bilaterally. Slit-lamp examination, intraocular pressure, and pupillary and fundus examinations were unremarkable. Cover testing revealed a small right hypertropia (Figure 1), measuring 3 prism diopters in dextroversion, 6 in primary gaze, and 8 in levoversion. The deviation increased to 10 prism diopters with right head tilt, consistent with a positive Bielschowsky test.

**Figure 2:**
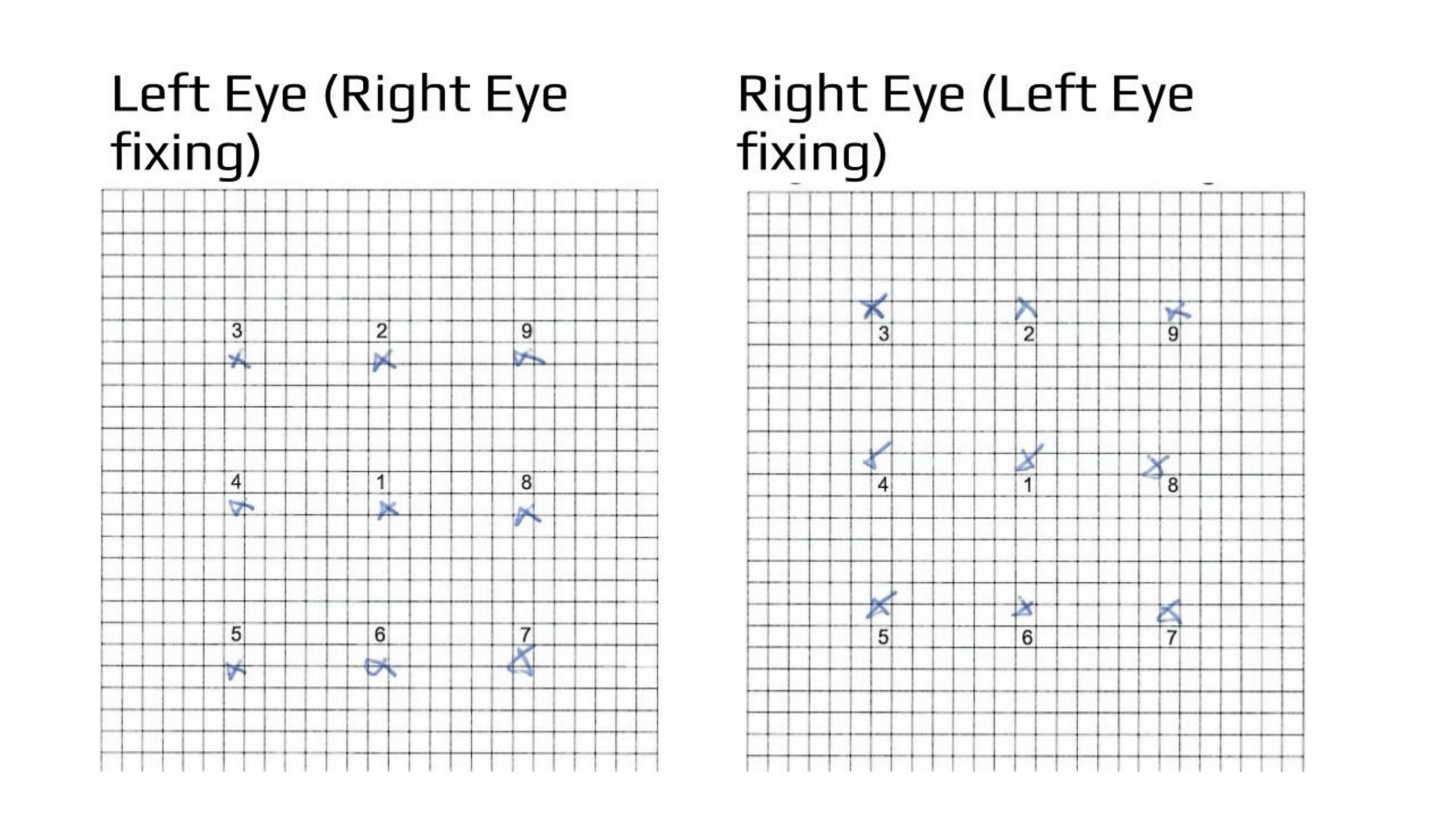
Ten days after hair replacement surgery, a Weiss screen revealed a small right hypertropia.

A Weiss screen revealed a similar deviation (Figure 2), with no significant torsion observed. Vertical fusion amplitude measured 2 prism diopters. The patient was diagnosed with acquired right fourth cranial nerve (trochlear) palsy. Given the minimal magnitude of the deviation and the absence of torsion, the prognosis was considered favorable. Consequently, a conservative “wait-and-see” approach was adopted, without prismatic correction.

**Figure 3:**
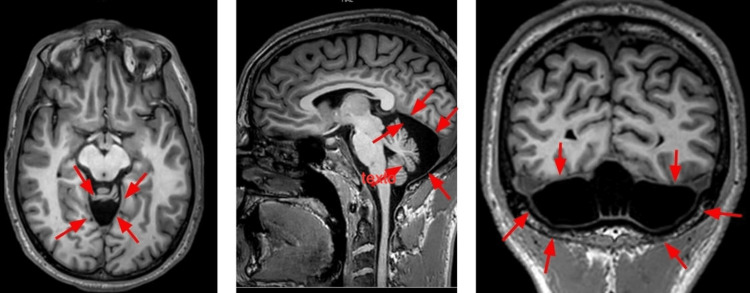
One month later, cerebral MRI demonstrated a mega cisterna magna (red arrows).

One month postoperatively, the deviation showed neither improvement nor worsening, and cerebral magnetic resonance imaging was unremarkable, except for the presence of a mega cisterna magna (Figure 3). At four months, the patient was free of diplopia in primary gaze, although vertical diplopia persisted in left gaze. By seven months, without any intervention, he was asymptomatic, and no deviation was observed in any gaze position.

## Discussion

We describe a neuro-ophthalmological complication of HRS, not previously reported, that started five days after surgery and we hypothesize that it could be related to a prolonged vertical position. We think that there may be a causal relationship as the diplopia occurred in a healthy patient and started five days after HRS. During this procedure, local anesthesia is injected into the scalp. While lidocaine has a proven toxic effect on extraocular muscles [11], this toxicity is not expected when injected at a distant site. Moreover, the timing (diplopia beginning five days post-surgery) does not support a causal relationship with the surgical procedure itself. Therefore we hypothesize that there may be a link between fourth nerve palsy and the prolonged vertical position that this patient maintained during the initial postoperative period.

The influence of gravity on cerebral and ocular physiology has been extensively studied in astronauts. These alterations, collectively referred to as spaceflight-associated neuro-ocular syndrome (SANS), have been the focus of intensive research in recent years [12]. Some studies have indicated that changes in resting posture may induce analogous physiological effects [13].

Posture has been implicated in ocular complications, particularly in specific clinical scenarios. As early as the 1950s, an association was established between prolonged prone positioning during spinal surgery and ocular complications [14]. Since then, several case series and reports have documented a link with non-arteritic ischemic optic neuropathy [15]. Other authors have described associations with vascular occlusions, orbital compartment syndrome, and angle-closure glaucoma [16,17].

It is well established that a lumbar puncture can produce a sixth cranial nerve paresis. The implicated mechanism is believed to involve a caudal displacement of the brainstem induced by changes in the pressure gradient. This displacement compresses and stretches the sixth nerve at the level of the petrous apex of the temporal bone [18,19]. The fourth cranial nerve, which encircles the brainstem, may be particularly susceptible to anteroposterior displacement of the brainstem [8-10].

Recent studies have demonstrated that transitioning from an upright to a prone position alters the direction of the gravitational vector and induces fluid shifts [20,21]. One month of bed rest at a 6° head-down tilt, combined with 0.5% atmospheric CO₂ - a spaceflight analog - resulted in a measurable enlargement of the cerebral ventricles [20]. Such postural changes may lead to cerebrospinal fluid (CSF) redistribution, which could be more pronounced or have more severe consequences in individuals with specific anatomical variations.

Mega cisterna magna is a cystic malformation of the posterior fossa, characterized by an enlarged cisterna magna, absence of hydrocephalus, and an intact cerebellar vermis. It is considered part of the Dandy-Walker malformation spectrum, which also includes classic Dandy-Walker malformation, Blake's pouch cyst, and posterior fossa arachnoid cyst [22]. Mega cisterna magna is generally regarded as an incidental finding; however, we propose that in this particular case it may have contributed to the development of the complication [22]. A pressure drop in a normally sized cisterna magna may induce minimal brainstem displacement, whereas in the presence of an enlarged cisterna magna, this movement may be amplified, resulting in greater instability.

Head trauma is the most common cause of acquired IV cranial nerve palsy in most case series [8]. Other etiologies include hydrocephalus, stroke, and tumors. The IV nerve possesses four unique characteristics: it is the longest and thinnest cranial nerve, its fascicle is dorsal, and it decussates within the brainstem. This anatomy renders it particularly susceptible to anteroposterior displacement resulting from trauma, as contrecoup forces may stretch the nerve and compress it against the free edge of the tentorium [8-10].

However, we acknowledge the limitations of our hypothesis. First, no similar cases have been reported in the existing literature. Second, there is an absence of dynamic studies demonstrating positional changes in the brainstem. Third, the lack of a prior ocular examination means that a preexisting congenital paresis cannot be definitively excluded. We consider a congenital paresis unlikely, given the absence of previous ophthalmologic symptoms, the complete normalization of extraocular motility, and the low amplitude of fusion. Nonetheless, only the publication of similar cases in the future will allow the proposed hypothesis to be tested.

## Conclusions

In conclusion, we report a rare case of diplopia following HRS, which we propose may have been triggered by a shift in CSF distribution in a patient with an unusual cerebral anatomical configuration. It has been demonstrated that lying down alters the gravitational vector and increases the volume of CSF-occupied spaces. Conversely, remaining upright (without lying down to sleep) may produce the opposite effect. We hypothesize that, in this particular case, a reduction in the volume of the mega cisterna magna may have resulted in an anteroposterior displacement of the brainstem, potentially stretching the fourth cranial nerve. Individuals with anatomical variants, such as the one described here, may be more susceptible than the general population to injury resulting from CSF redistribution.
